# Advances in the Research on Anticardiolipin Antibody

**DOI:** 10.1155/2019/8380214

**Published:** 2019-12-01

**Authors:** Dan Wang, Wenxin Lv, Shichang Zhang, Jiexin Zhang

**Affiliations:** Department of Laboratory Medicine, The First Affiliated Hospital of Nanjing Medical University, 210029 Nanjing, China

## Abstract

Anticardiolipin antibody (ACA) is a kind of autoantibody and is one of the antiphospholipid antibodies (aPLs). Phospholipids with a negative charge on platelets and endothelial cell membranes are ACA target antigens. ACA is common in systemic lupus erythematosus and other autoimmune diseases and is closely associated with thrombosis, thrombocytopenia, and spontaneous abortion. In 1983, Harris established a method for detecting ACA, and research on the antibody has gained worldwide attention and has developed rapidly. For this review, we browsed articles that cover most of the ACA-related studies in the last 25 years and extracted influential ideas and conclusions in this field.

## 1. Introduction

The first antiphospholipid antibody (aPL) was found in patients with syphilis in 1906, and its associated antigens were identified as phospholipids. The aPLs including anticardiolipin antibody (ACA), anti-beta2-glycoprotein I (*β*_2_GPI), and lupus anticoagulant (LA) are a heterogeneous group of autoantibodies reacting against phospholipids, phospholipid-protein complexes, and phospholipid-binding proteins [1]. In 1941, cardiophospholipid was isolated from the heart of the cow and named, which has the highest concentration in the mammalian myocardium and skeletal muscle. Each cardiac phospholipid molecule contains four unsaturated fatty acids, which are easily oxidized or polymerized. The study found that ACA had cross-reactivity with most negatively charged phospholipids. ACA can be combined with negatively charged phosphodiesters in cardiac phospholipid molecules, and the fatty acids in phospholipid molecules are essential components of their antigenicity.

## 2. Brief Introduction of Anticardiolipin Antibody

ACA is a group of heterogeneous antibodies that are classified into IgG, IgA, and IgM. ACA can be combined with cardiac phosphatide, phosphatidyl serine, or phosphatidyl inositol. The target antigen of ACA is controversial. It can be phospholipids in the heart; phospholipid-binding proteins in the plasma, such as *β*_2_GPI; or protein and phospholipid compounds. The target antigen may also be the phospholipids that are exposed by the conformation change after the formation of the complex or the new antigenic determinants on *β*_2_GPI [2]. ACA can be detected in many diseases, including autoimmune disease, recurrent abortion, cerebrovascular disease, and infectious disease. The relationship between ACA and its clinical manifestation is divided into two categories. ACA in autoimmune disease and antiphospholipid syndrome (APS) is within the autoimmune range, and its target antigen is phospholipid-binding protein, which can cause coagulation disorder; syphilis and other infectious diseases contain nonimmune ACA, whose target antigen is cardiophosphate, and it does not depend on plasma proteins such as *β*_2_GPI.

## 3. Clinical Laboratory Detection Assay

In 1983, Harris et al. first established the ACA test by using cardiolipin as an antigen, a mixture of gelatin/phosphate-buffered saline (PBS) to dilute patients' serum samples and radiolabeled antihuman IgG or IgM to detect bound ACA as a radioimmunoassay. Over the next few years, the test method evolved so that fetal cow serum or adult bovine serum replaced gelatin/PBS as a sample diluent to provide sufficient *β*_2_GPI for a valid test, enzyme-labeled antihuman IgG or IgM antibodies replaced radiolabeled antihuman IgG or IgM antibodies, and ACA enzyme-linked immunosorbent assay (ELISA) was established [3]. ACA from patients with APS requires *β*_2_GPI as a cofactor for cardiolipin binding [4]. Thus, ACA assays may indirectly be dependent on the function and structure of *β*_2_GPI.

Many scholars have been working on the improvement and standardization of ELISA detection methods. It was demonstrated that both discrepancies and the lack of interlaboratory agreement are mainly due to the way that laboratories perform the test, the way that the test is calibrated, and how the results are calculated. When laboratories use the standard ELISA kit and calculate in a uniform manner, the agreement between laboratories gains much improvement [5]. At the 8^th^ International Antiphospholipid Antibody Symposium, experts pointed out that the routine use of an ELISA kit was the first choice for the diagnosis of APS. However, no standardized ELISA methodology is available and intra- and interlaboratory variability remains at a high level.

Recently, new methods have been proven to be useful for detecting ACA. Line immunoassays employing a novel hydrophobic solid phase for the detection of ACA seem to be an intriguing alternative [6, 7]. Chemiluminescence immunoassay (CLIA) has been available for ACA detection since 2010. A fully automated CLIA can use paramagnetic particles coated with cardiolipin or human *β*_2_GPI to capture antibodies from the serum samples and then detect ACA. Meneghel et al. showed that CLIA had a significantly lower comparative sensitivity for IgM ACA but a significantly higher comparative specificity for IgM ACA with respect to a homemade ELISA [8]. Fluorescence enzyme immunoassay, which measured the fluorescence in the reaction mixture once the reaction was stopped, was compared with homemade ELISA. Mattia et al. found that the sensitivities of the two methods were similar with the exception of IgM ACA [9]. Collectively, fully automated systems of ACA assay should be developed for standardizing ACA testing as a result of the significant intra-assay and intra-assay variation.

## 4. The Clinical Significance of ACA Laboratory Detection

### 4.1. Autoimmune Disease

#### 4.1.1. Systemic Lupus Erythematosus

Systemic lupus erythematosus (SLE) is a chronic, multisystemic, autoimmune inflammatory disorder that primarily affects premenopausal women [10]. In 2012, the Systemic Lupus International Collaborating Clinics group proposed new classification criteria that include clinical and serological contents to diagnose and classify SLE, and ACA is one of the six serological contents [11]. Hobbs et al. conducted a single-center, retrospective inception cohort study to evaluate clinicopathological features, including the ACA of pediatric patients with new-onset SLE nephritis. They found that 90% (19/21) of the patients were positive for ACA [12]. In another study including 390 SLE patients, 47% had an elevated level of ACA. SLE patients had prolonged activated partial thromboplastin time (APTT), thrombocytopenia, and positive Coombs' test results, but they did not have APS. Prolonged APTT was strongly associated with venous or arterial thrombosis [13]. A study found that platelet activation was not induced by ACA+LA (lupus anticoagulant)+plasma only but was significantly augmented by ACA+LA+ plasma in combination with adenosine diphosphate (ADP) at a low concentration that had only a modest effect on platelet activation. In contrast, none of the ACA+LA-, ACA-LA+, or ACA-LA- plasma samples were capable of enhancing platelet activation even in the presence of ADP stimulation, indicating that ACA and LA may cooperate to promote platelet activation and may be involved in the pathogenesis of arterial thrombosis and thrombocytopenia in patients with SLE [14]. Alharbi et al. evaluated the differences between systemic sclerosis systemic lupus erythematosus overlap syndrome (SSc-SLE) and SLE and found that the level of ACA was increased in patients with SSc-SLE [15].

#### 4.1.2. APS

APS is an autoimmune disorder that is clinically characterized by recurrent venous and/or arterial thromboembolic events or pregnancy morbidity and is associated with aPLs [16–18]. Patients must have both clinical and laboratory criteria to be diagnosed with APS, and APS diagnosis requires the presence of at least one of the three aPLs [19]. Vascular thrombosis is defined as one or more clinical episodes of arterial, venous, or small-vessel thrombosis, involving any organ, and is confirmed by appropriate imaging and/or histopathological analyses [19]. The clinical classification criteria of pregnancy morbidity are as follows: (1) one or more unexplained deaths of a morphologically healthy fetus at or beyond the 10^th^ week of gestation, with normal fetal morphology; (2) one or more premature births of a morphologically healthy neonate at or before the 10^th^ week of gestation because of eclampsia or severe preeclampsia, or placental insufficiency; and (3) three or more unexplained consecutive spontaneous abortions before the 10^th^ week of gestation, with maternal anatomic or hormonal abnormalities, in which maternal or paternal chromosomal abnormalities have been excluded. The updated Sydney classification scheme recommends (1) a lupus anticoagulant detection according to guidelines published by the International Society on Thrombosis and Hemostasis; (2) ACA (IgG or IgM) exceeding 40 IgG or IgM antiphospholipid units; or (3) anti-*β*_2_GPI antibodies (IgG or IgM) at levels exceeding the 99^th^ percentile measured by ELISA [20]. It was reported that ACA is positive in 84-90% of APS patients and in 25% of APS patients (without LA) [21]. The importance of ACA testing in the diagnosis of APS was challenged in 2002; however, Nash et al. demonstrated that the omission of ACA testing from the clinical investigation of APS could lead to a failure to diagnose the syndrome in a large proportion of patients. In their report, using 123 patients with persistent aPL, more than one-quarter of the cohort of patients' studies were positive for ACA and negative for LA and anti-*β*_2_GPI [22]. Thus, the importance of ACA in the diagnosis of APS cannot be ignored.

#### 4.1.3. Rheumatoid Arthritis

Rheumatoid arthritis (RA) is a connective tissue disease that influences organs of the whole body. Articular inflammation is the most prominent manifestation, and skin involvement is often seen. Serologically, rheumatoid arthritis may show rheumatoid factor (RF) and/or other autoantibodies. Wolf et al. collected specimens from 173 consecutive patients with rheumatoid arthritis and analyzed the laboratory results and clinical manifestations. Abnormally elevated IgG and/or IgM ACA levels were detected by ELISA in the sera of 55 (32%) patients. They also found that of 36 patients with rheumatoid nodules, 17 had positive ACA levels (sensitivity 47%) indicating a statistically significant association [23]. In contrast, Bobbio-Pallavicini et al. investigated the effect of long-term infliximab treatment on various autoantibodies in patients with RA. The results showed an increase in the ACA titer without any related clinical manifestations [24]. Seriolo et al. suggested that the association between ACA positivity and decreased levels of free protein S in RA patients may represent one of the risk factors for thrombotic events [25].

#### 4.1.4. Immune Thrombocytopenic Purpura

Immune thrombocytopenic purpura (ITP) is an acquired disorder characterized by isolated thrombocytopenia resulting from autoantibody-mediated peripheral platelet destruction and the absence of any obvious initiating and/or underlying cause of thrombocytopenia. Despujol et al. collected data from 215 patients with ITP and found that 54 (25%) patients had ACA, including 42 (20%) that were IgG-ACA positive [26].

Thrombocytopenia, as a manifestation of primary APS, has a reported prevalence of 20 to 46%. Although evidence suggests that ACA may bind activated platelet membranes and cause platelet destruction, the pathogenesis of thrombocytopenia related to ACA remains unclear. Carlos et al. detected the IgG and IgM of aPLs via ELISA in 21 patients with ITP and 33 with APS. The results showed that the frequencies of IgG antibodies in ITP were ACA (47.6%) > anti-*β*_2_GPI (19%). In APS, conversely, anti-*β*_2_GPI was the most frequent (73%) [27].

### 4.2. Thrombosis

ACA is one of the indicators of thrombosis. Naess et al. suggested that elevated ACA levels were not a risk factor for subsequent venous thrombosis in a general population via a large population-based nested case-cohort study containing 508 venous thrombosis cases and 1464 matched control subjects from a total 66 140 participants. Elevated ACA levels were still associated with a twofold increased risk of venous thrombosis in the presence of autoimmune disease (mainly systemic lupus erythematosus) [28]. Godoy et al. found that the serum ACA levels of 34 patients who suffered from deep vein thrombosis were above the normal reference range. However, in 6 patients with recurrent thrombosis, 4 tested as positive. Thus, patients with deep vein thrombosis who are positive for ACA had a higher risk of recurrent thrombosis [29].

ACA attracts monocytes into the vessel wall and induces their adherence to endothelial cells, which is mediated by adhesion molecules such as intercellular cell adhesion molecule-1, vascular cell adhesion molecule-1, and E-selectin [30]. A study confirmed that ACA derived from (NZW × BXSB) *F*_1_ mice, a model of antiphospholipid syndrome with myocardial infarction, can cross-react with oxidized low-density lipoprotein, which is believed to induce the transformation of macrophages into foam cells and, in some cases, to cause endothelial cell damage [31]. Another study found that IgG ACA was able to enhance the expression of monocyte chemotactic protein-1 (MCP-1) at both the protein and mRNA levels, and the overexpression of MCP-1 has been implicated in several pathological conditions, including atherosclerosis, thrombosis, and inflammatory disease [32]. Alves et al. revealed that ACA induces nitric oxide production acutely through the increased expression of inducible nitric oxide synthase in both *ex vivo* and *in vivo* models which might lead to feedback inhibition of endothelium-derived nitric oxide and increase the risk of thrombosis [33]. The possible mechanisms of ACA causing thrombosis are as follows: (1) ACA reacts with the membrane phospholipids of platelets or vascular endothelial cells, thereby inhibiting the synthesis of prostacyclin in vascular endothelial cells (PGI_2_). Thus, the factors contributing to thrombosis are increased [34]. (2) After ACA damages vascular endothelial cells, the release of plasminogen activator is reduced, thereby increasing the tendency of thrombosis [35]. (3) ACA-IgG can also cause direct immune damage to endothelial cells, triggering platelet adhesion, aggregation, and the activation of factor XII [36]. (4) ACA can inhibit thrombin regulation, reduce the activation of protein C, and increase blood coagulation activity *in vivo*, thereby promoting thrombosis.

### 4.3. Recurrent Abortion

Recurrent abortion refers to consecutive spontaneous abortions in women. A study aimed at evaluating the prevalence of high ACA in women with histories of at least two miscarriages found that high ACA levels were identified in 55.77% of the individuals. A systematic review and meta-analysis demonstrated a positive association between antiphospholipid antibodies and/or APS in patients with recurrent abortion [37]. A study analyzed 85 antenatal patients with recurrent fetal loss (cases) and an equal number of antenatal patients without recurrent fetal loss (control) matched for age. The conclusion was that the prevalence of aPL among antenatal patients with recurrent abortion was at least 3 times higher than that of the normal antenatal clients [38]. Previous studies showed that triple aPL positivity (ACA, anti-*β*_2_GPI, and LA) is associated with pregnancy complications in aPL carriers [39, 40]. The above experimental data confirmed a positive correlation between ACA and recurrent abortion, especially in relation to late-stage recurrent abortion. Therefore, ACA can be used as one of the indicators to predict the abortion in high-risk women.

The possible mechanism of ACA resulting in recurrent abortion includes the following aspects: (1) ACA interferes with calcium-dependent phospholipid-binding protein V, which affects the flow of blood between the villi [41]. (2) The combination of ACA and vascular endothelial phosphatide can damage the vascular endothelium and cause local thrombus formation, resulting in insufficient blood supply for the decidual membrane and placenta, vascular lesions, placental embolism, and infarction. (3) ACA reacts with platelets or the membrane phospholipids of vascular endothelial cells, causing local blood vessels to contract, platelet aggregation, and a decreased blood volume of the placenta, finally resulting in a pathological pregnancy [42]. (4) The serum total complement level decreased in APS patients, and the circulating immune complex increased. There is excessive activation of complement, which leads to fetal abortion and limited embryo development [43]. (5) In addition, ACA can cause placental vasculitis, which results in inadequate fetal oxygen supply and nutrition, causing fetal distress and death.

### 4.4. Cerebrovascular Disease

ACA is associated with cerebrovascular disease. Epidemiological studies of patients with acute nonhemorrhagic cerebral apoplexy showed that ACA was significantly elevated in patients with acute cerebral infarction and had increased before onset. ACA may be involved in the process of cerebral infarction. Studies have shown that the ACA level of multifocal cerebral infarction patients is significantly higher than that of patients with single cerebral infarction [44]. Cerebral infarction patients positive for ACA had a significantly increased risk of a second cerebral infarction. Therefore, ACA can provide a reference indexes for clinical treatment [45].

The relationship between ACA and cerebrovascular disease risk factors is generally considered as follows: (1) Age: in recent years, it is believed that ACA has a higher positive rate in young patients with cerebral infarction [46]. Therefore, in middle-aged and young people, if there is unexplained cerebral apoplexy, transient ischemic attack, deep vein thrombosis, etc., it can be further examined by analyzing ACA. A study of ACA-positive patients with cerebral infarction found that there were more female patients than males. Positive patients were more likely to have heart disease, blood disease, and neurological complications. In addition to other risk factors, the recurrence rate for female patients was higher than that for male patients [47]. (2) Diabetes: patients with diabetes have a disorder of glucose metabolism and lipid metabolism that can produce a large number of oxygen free radicals, which causes the vascular endothelial cells to be damaged, so that the platelet function is hyperactive, producing a large amount of ACA. ACA, in turn, affects the synthesis of PGI2 by vascular endothelial cells, interfering with thrombus adjusting element, fibrinolysis enzyme activator, and the activity of the protein C system and inhibiting thrombin III activity. In addition, ACA also promotes the activation of platelet function and microvascular disease, thereby involving thrombosis in the body. (3) High blood pressure: hypertension itself can lead to vascular endothelial cell injury. ACA can react with the membrane phospholipids in the endothelial cell membrane of the arterial wall, and the damage to the endothelial cells leads to the exposure of membrane phospholipids, inducing the production of ACA. On the other hand, ACA aggravates the damage to the endothelium, thus forming a vicious circle. (4) Smoking: the ACA of smokers is significantly higher than that of nonsmokers, and its mechanism may be as follows: smoking can cause LDL to be susceptible to oxidation, leading to an increase in oxidative products in the body. In addition, the synthesis and activity of superoxide dismutase and glutathione peroxidase can be reduced. Oxygen free radicals increase greatly, causing vascular endothelial cell injury to induce the production of ACA [48]. (5) Hyperlipidemia: the combination of ACA and the compound product of *β*_2_GPI and phospholipid will reduce the removal of triglycerides, leading to hypertriglyceridemia [49]. At the same time, due to the increased body fat, the body will produce a large number of oxide free radicals and oxidation products and will also promote the formation of mural thrombi, thus causing the occurrence of cerebrovascular accidents and cerebral infarction.

### 4.5. Infectious Diseases and Other Symptoms

Since the association between aPL and syphilis was first described, many other viral, bacterial, and parasitic infections have been shown to induce antiphospholipid antibodies, notably ACA. A review of the literature shows that while ACA occurs frequently in viral infections, particularly in HIV (49.75%), HBV (24%), and HCV (20%), it is not correlated with thrombosis risk or hematological manifestations. Concerning bacterial infections, ACA is often present in leprosy (42.7%) [50].

The possible mechanism of infectious diseases inducing ACA is still not very clear, but molecular mimicry has been studied. Barton demonstrated that the two most broadly reactive HIV-1 envelope gp41 human monoclonal antibodies, 2F5 and 4E10, are polyspecific autoantibodies reactive with the phospholipid cardiolipin. ACA may be induced by autoantigen mimicry of the conserved membrane-proximal epitopes of the virus [51]. A previous study showed that immunological cross-reactivates between autoantigens and viruses, molecular mimicry, and immunomodulation by viral proteins may account for both cross-reactivity with autoantigens and abnormal T and B cell functions in autoimmune disorders [52].

A few articles studied ACA in other diseases, including epilepsy, preeclampsia, chorea, Budd-Chiari syndrome, and chronic pancreatitis (especially for autoimmune pancreatitis). There might be an autoimmune-mediated pathogenesis, especially via ACA, in the onset of these diseases [53–57].

## 5. Conclusion

We believe that with the continuous improvements in medicine and the rapid development of science and technology, the detection method of ACA will be more standardized. Considering that phospholipids with negative charge are the main constituents of the cell membrane and are widely distributed throughout the body, ACA may play a role in some stages of the pathogenesis of several diseases. These antibody/antibody complexes represented by ACA are mostly involved in complex systemic diseases involving multiple organs (SLE, thrombotic diseases, cardiovascular diseases, etc.). Because of this, insufficient research has been conducted on its molecular mechanism. We hope that through future research, we can find a messenger (such as effector cells) to organically link the “organ-antibody axis” and investigate ACA-related diseases from a new perspective. As a result, the study of the nature of ACA, its target antigen, and the method of ACA laboratory testing will help to advance the modern precision medicine.
